# Dietary-related characteristics and cataract risk: evidence from a mendelian randomization study

**DOI:** 10.3389/ebm.2025.10544

**Published:** 2025-08-04

**Authors:** Chen Li, Yicheng Lu, Mingxuan Chen, Qing Zhang, Zhe Zhang, Wenqun Xi, Weihua Yang

**Affiliations:** ^1^ Department of Ophthalmology, The First Affiliated Hospital of Soochow University, SuZhou, Jiangsu, China; ^2^ School of Clinical Medicine, Medical College of Soochow University, SuZhou, Jiangsu, China; ^3^ Shenzhen Eye Hospital, Shenzhen Eye Medical Center, Southern Medical University, Shenzhen, China

**Keywords:** cataract, dietary-related characteristics, mendelian randomization, causal relationship, prevention strategy

## Abstract

Cataract is the leading cause of blindness globally, imposing a significant socioeconomic burden. While diet is associated with various eye diseases, the causal relationship between dietary-related characteristics (DRCs) and cataract remains unclear. This study investigates the causal associations between DRCs and cataract using Mendelian randomization (MR) to provide insights into potential dietary interventions for cataract prevention. We conducted a two-sample MR analysis using data from the open GWAS database, focusing on individuals of European descent. Instrumental variables were selected based on stringent criteria, and multiple MR methods were applied to estimate causal effects. Sensitivity analyses assessed the robustness of the findings. Significant causal associations were found between oily fish intake (OR = 0.86) and variation in diet (OR = 1.26) with cataract. Sensitivity analyses supported the robustness of these findings. Mediation effect analysis suggested that the intake of oily fish might indirectly influence cataract risk through metabolites. This study provides evidence for causal relationships between specific DRCs and cataract, highlighting the potential role of dietary interventions in cataract prevention.

## Graphical Abstract

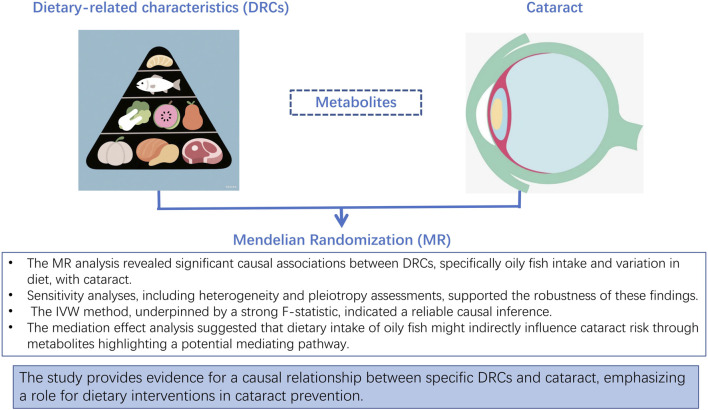

## Impact statement

This study provides the first Mendelian randomization evidence for causal relationships between oily fish intake, dietary variation, and cataract, enabling targeted dietary strategies for high-risk populations.

## Introduction

Cataracts are a leading cause of blindness globally, affecting approximately 17.01 million people and causing severe visual impairment in 83.48 million individuals by 2020. They impose a substantial burden on patients and society [1]. Aging is the primary cause of cataract development, but genetic predisposition, environmental factors, and lifestyle choices also increase the risk. Currently, cataracts are mainly treated surgically, which involves risks and economic burdens. There are no effective medications to prevent or treat moderate to severe cataracts [2].

Dietary intake is associated with various eye diseases, including cataracts [3–7]. Studies have shown that a high intake of carbohydrates and polyunsaturated fatty acids increases the risk of cortical and nuclear cataracts, while a high intake of protein, especially animal protein, has a protective effect against posterior subcapsular cataracts [6]. Compared with those who consume large amounts of meat, vegetarians have a lower risk of cataracts, indicating that plant-based diets are protective [8]. The dietary pattern of “dairy and vegetables” and traditional dietary patterns are negatively correlated with the occurrence of cataracts. In contrast, high-carbohydrate and monosaccharide diets increase the risk [9]. Dietary factors may influence cataracts by affecting lens oxidative stress, and higher antioxidant intake may reduce the incidence and severity of cataracts [10]. However, the causal relationship between diet and cataracts remains unclear.

In this study, we used data from the GWAS database and employed a two-sample Mendelian randomization (MR) approach to investigate the causal relationship between dietary-related characteristics (DRCs) and cataract risk, focusing on individuals of European descent. Our analysis included selecting single nucleotide polymorphisms (SNPs) associated with DRCs as instrumental variables (IVs), using various MR methods to estimate causal relationships, and conducting sensitivity analyses to ensure robustness. This study aims to identify dietary factors that influence cataract risk and provide strategies for dietary interventions and non-surgical treatments.

## Materials and methods

### Data sources

**FIGURE 1:**
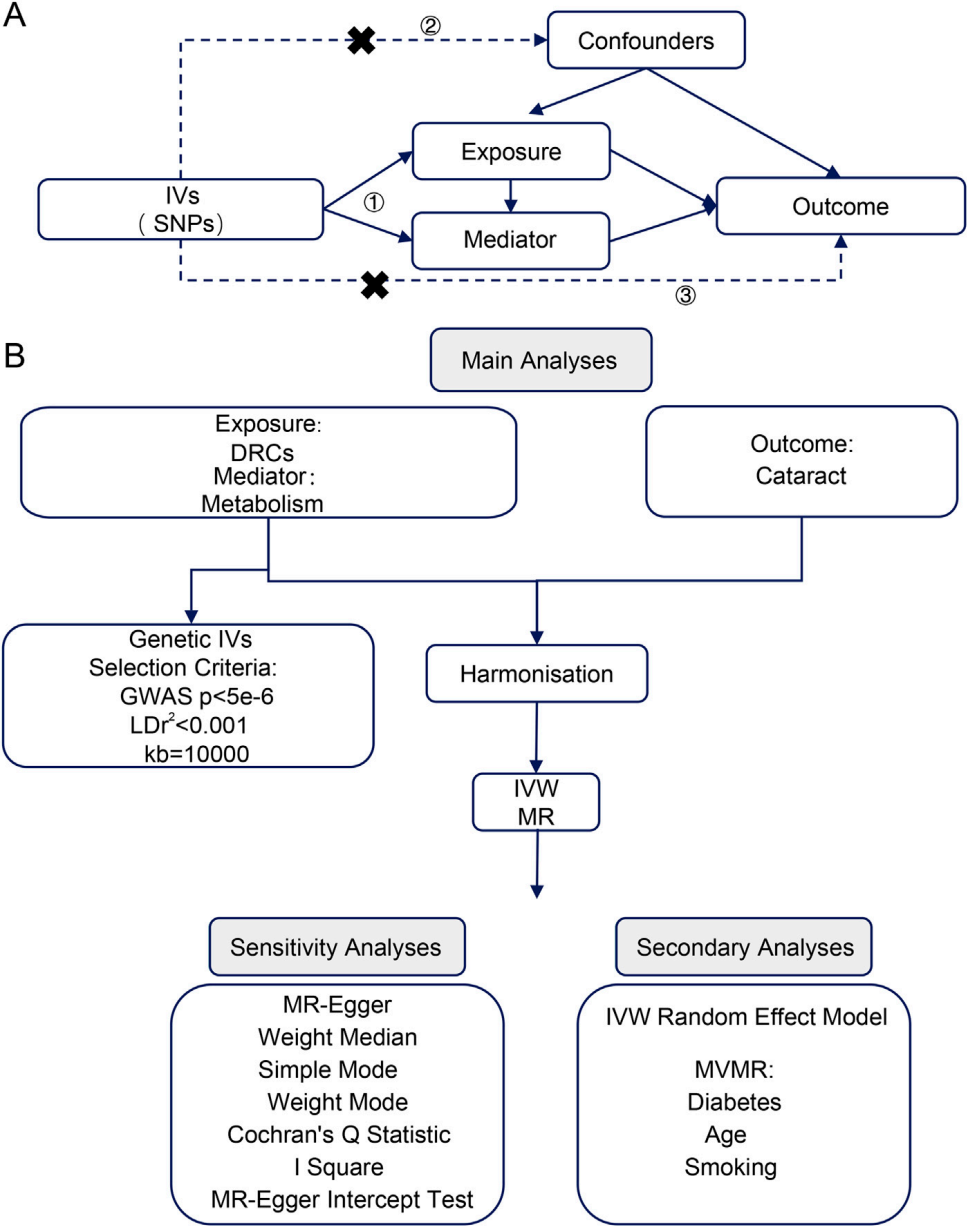
**(A)** Key assumptions of Mendelian randomization analysis. **(B)** Flowchart of the analytical methodology in this study. IVs, instrumental variables; SNPs, single nucleotide polymorphisms; DRCs, dietary-related characteristics; IVW, inverse variance weighted; MR, Mendelian randomization; GWAS, genome wide association study; LD, linkage disequilibrium; MR-Egger, Mendelian randomization-Egger. MVMR, multivariable Mendelian randomization.

All data in this study were obtained from publicly available databases. The GWAS data were downloaded from the Open GWAS database^1^ [11]. GWAS data for dietary-related characteristics were acquired by searching for the keywords “diet” or “intake” and were derived from European ancestry samples. The relevant data are in Supplementary Table S1. The cataract GWAS data (ebi-a-GCST90018814) included 39,519 cases and 452,358 controls, also based on European ancestry populations. We identified cataract risk factors through a literature review and obtained data for diabetes, age, and smoking [12–14], all based on European samples. Additionally, we acquired data for 249 metabolites by searching for “met-d,” selecting European individuals, and presented the data in Supplementary Table S2. Table 1 provides an overview of the GWAS data characteristics, including population information, sample size, sources, and validation approaches. The report follows the STROBE-MR Statement guidelines. This study uses open-source data, so there are no ethical issues or conflicts of interest. The study design is shown in Figure 1.

**TABLE 1:** Data sources and characteristics.

Data type	Description	Sample size	Source	Validation method
DRCs	Genetic variants associated with dietary habits (e.g., “Oily fish intake”; “Variation in diet”)	Oily fish intake (N = 460,443); Variation in diet (N = 460,884)	Open GWAS database	GWAS data analysis
Cataracts	Case-control data for cataracts derived from GWAS database (GBE ID: ebi-a-GCST90018814)	39,519 cases and 452,358 controls	Open GWAS database	GWAS data analysis
Covariates (e.g., diabetes, age, smoking)	Data sources and validation methods for covariates including diabetes, age, and smoking	finn-b-DIABETES_FG (218,792 (36,219 cases and 182,573 controls)); ukb-b-8727 (N = 170,248); finn-b-SMOKING (138,088 (1321 cases and 136,767 controls))	Literature and public databases	Literature review and GWAS data analysis

### Instrument selection and strength assessment

Initially, SNPs from the exposure GWAS with a statistical significance threshold of P < 5 × 10^-6^ were considered, prioritizing those with the strongest evidence of association. Additionally, we identified SNPs in linkage disequilibrium. To mitigate the potential bias introduced by weak IVs, the F-statistic was employed as a quantitative measure of instrument strength. An F-statistic threshold of 10 was established and the formula for the F-statistic is as follows:

$$F=\frac{N-k-1}{k}\times \frac{R^{2}}{1-R^{2}}$$

Where *R* ^2^ is the coefficient of determination from the regression of the exposure on the IV, k denotes the number of IV used, and N is the sample size.

### MR causal effect estimation

Multiple two-sample MR methods were employed to estimate the causal effect of DRCs on cataract, including IVW, MR-Egger, weighted median, simple mode, and weight mode. Besides, we use the Steiger directionality test from the TwoSampleMR package to determine the direction of causality. The same methods are applied to assess the potential causal effect of the outcome on the exposure for reverse causality.

### Sensitivity analysis

Sensitivity analysis includes heterogeneity assessment, pleiotropy assessment, and leave-one-out analysis. First, we used the Cochran Q test to evaluate the heterogeneity between SNP estimates. The statistically significant result of the Cochran Q test suggests the presence of significant heterogeneity in our analysis results. We further quantified the proportion of heterogeneity using the I^2^ statistic. The calculation formula for I^2^ values is as follows:

$$I^{2}=\frac{Q-df}{Q}\times 100\%$$

Subsequently, we employed the MR-Egger method to test for pleiotropy in the IVs. In addition, by sequentially removing individual SNPs and calculating the MR results for the remaining IVs, we assessed whether the SNP affects the association between DRCs and cataract.

### Multivariable mendelian randomization (MVMR) analysis and mediation effect estimation

We conducted individual and overall MVMR analyses on cataract-related risk factors, such as diabetes, age, and smoking, along with DRCs obtained post-screening, to determine the direct effects of these DRCs on cataract. The mediation effect’s estimates and standard errors are calculated using the following formula:

$$\beta_{M}=\beta_{A}\times \beta_{B}$$

$${SE}_{M}=\sqrt{{\left(\beta_{A}\times {SE}_{B}\right)}^{2}+{\left(\beta_{B}\times {SE}_{A}\right)}^{2}+{{SE}_{A}}^{2}\times {{SE}_{B}}^{2}}$$

*β*_M_ represent the mediated effect size, *β*_*A*_ represent the MR effect of DRCs on metabolites, *β*_*B*_ represent the direct effect of metabolites on cataract obtained via MVMR, *SE*_*M*_ represent the standard error of the mediation effect, *SE*_*A*_ represent the standard error of the MR analysis for DRCs on metabolites, and *SE*_*B*_ represent the standard error of the MR analysis for metabolites on cataract.

### Statistical analysis

All computations and statistical evaluations were conducted utilizing R (version 4.2.2) [15]. In the context of MR analysis examining the effects of exposures on outcomes, OR and 95% confidence intervals served as the primary metrics for evaluation. All statistical P-values were obtained from two-tailed tests; SNPs identified in GWAS were deemed statistically significant at a threshold of P < 5 × 10^-6^, whereas other statistical assessments were considered significant at P < 0.05.

## Results

### Tool variable selection

Figure 1 illustrates the technical roadmap of this study. Based on the selection criteria for IVs in this study, SNPs with linkage disequilibrium were excluded, and SNPs related to DRC that matched the GWAS data for cataract were included as IVs. Supplementary Table S3 presents the outcomes of the IV selection for each indicator, highlighting only those indicators that exhibited significant results in the MR analysis. The F-statistic associated with the IVs for these indicators exceeds the threshold of 10, suggesting that the majority of SNPs identified in this study function as robust effect IVs.

### Estimation of causal effects

**FIGURE 2:**
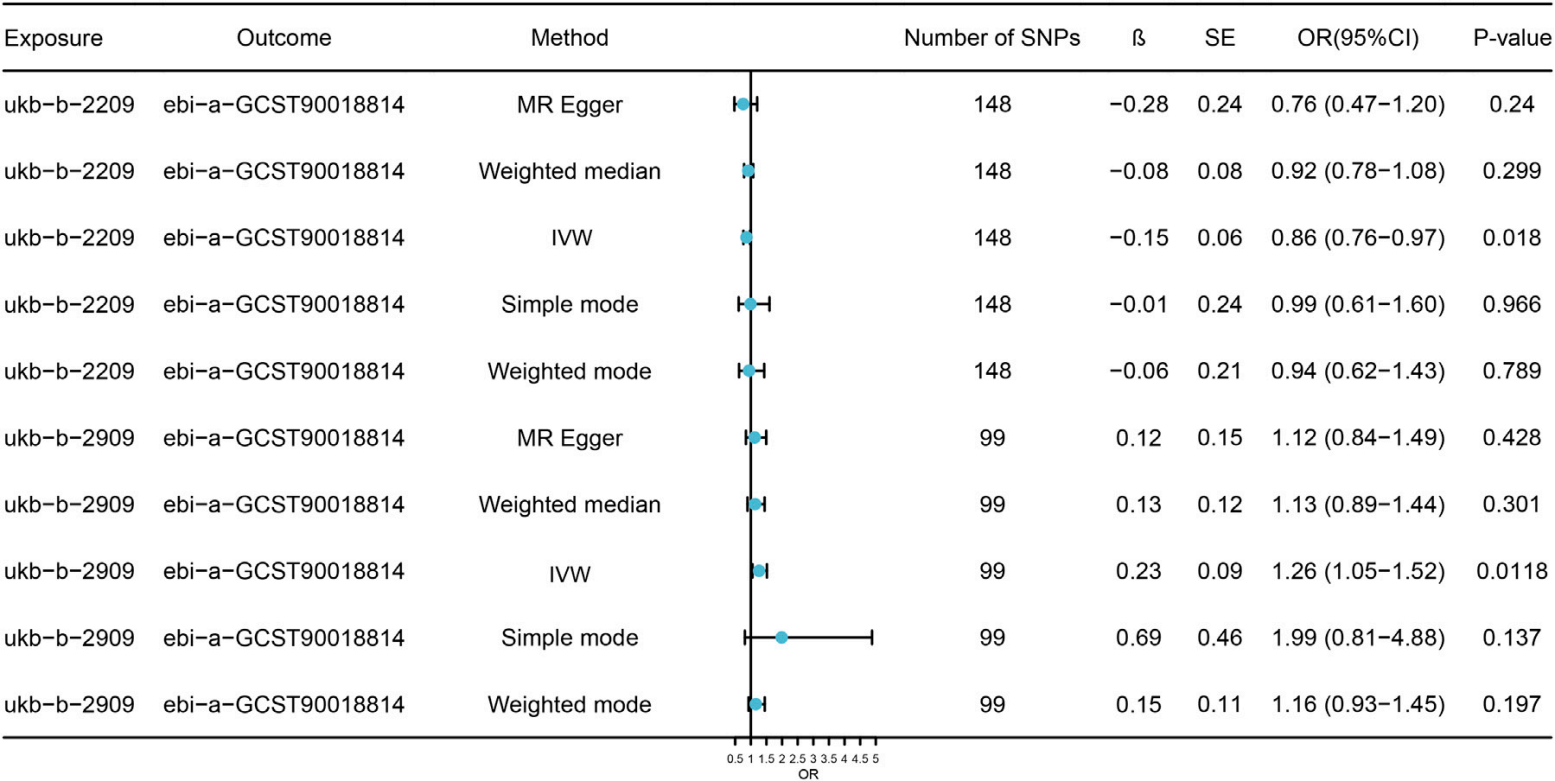
Model analysis results of the MR analysis of DRCs and cataract. Forest figure displays MR model related to DRC and the causal relation analysis result of cataract, effect evaluation value with the OR and 95% CI show, at the same time show the model using IV number and calculating the Beta values and standard error. SNPs, single nucleotide polymorphisms; SE, standard error; OR, odds ratio; CI, confidence interval; β, Mendelian randomization analysis effect coefficient; IVW, Inverse variance weighted.

**FIGURE 3:**
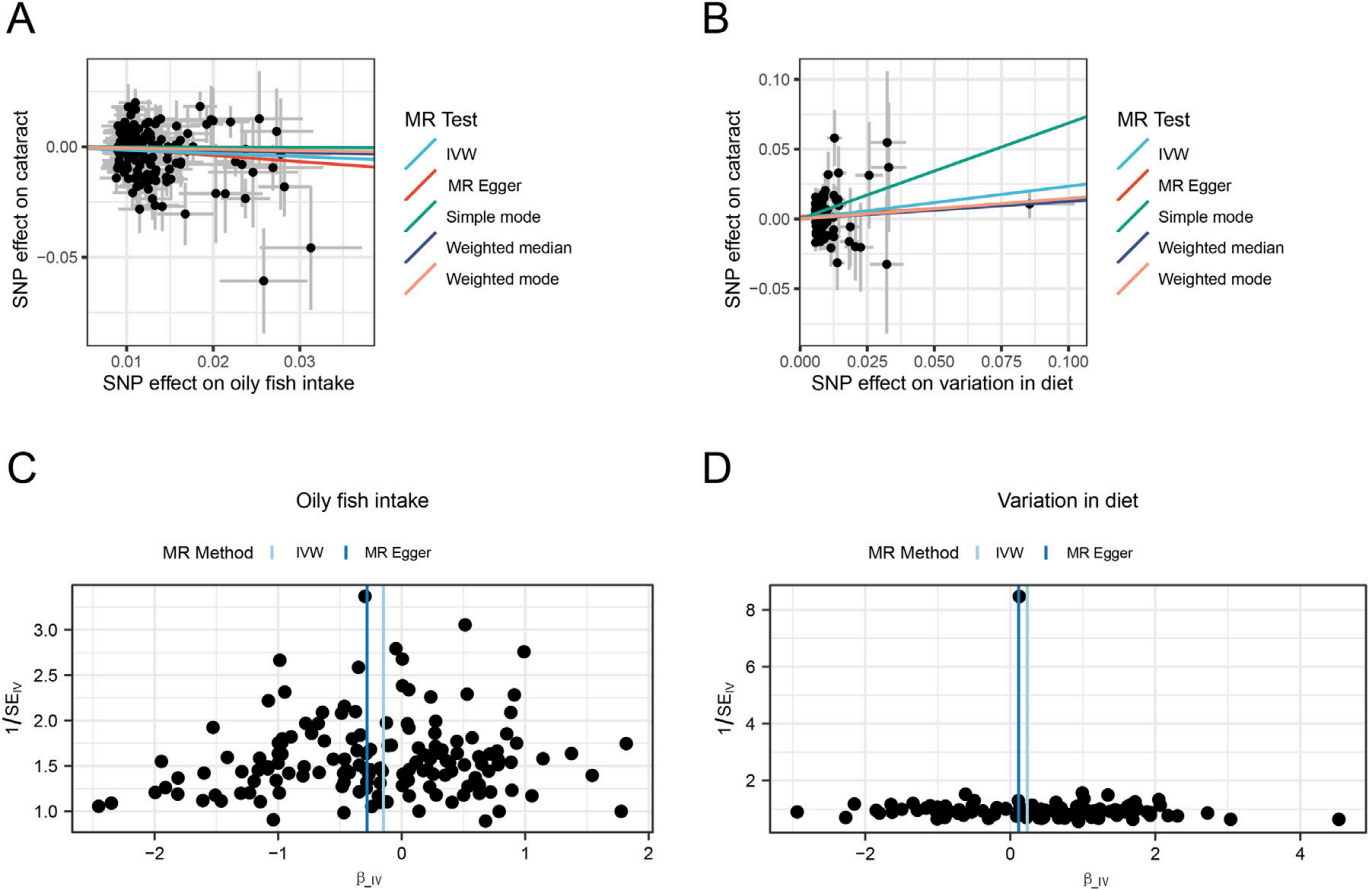
MR analysis of the causal relationship between DRCs and cataract. **(A,B)** Scatter plot showing the causal relationship between dietary patterns and cataract, with the slope of the line indicating the magnitude of the causal relationship predicted by the different models; **(C,D)** Funnel chart showing dietary pattern and causation of cataract. SNPs, single nucleotide polymorphisms; SE, standard error; OR, odds ratio; CI, confidence interval; IVW, Inverse variance weighted.

The IVW model results for the relationship between DRCs and cataract indicate that oily fish intake (OR = 0.86, 95%CI: 0.76–0.97) and variation in diet (OR = 1.26, 95%CI:1.05–1.52), two DRCs have a significant causal association with cataract. These results indicate that increased intake of oily fish is associated with a reduced risk of cataracts, suggesting a protective effect. In contrast, greater dietary diversity is significantly associated with an elevated risk of cataracts, with statistical significance (Supplementary Table S4; Figure 2). Funnel plots (Figures 3A,B) and scatter plots (Figures 3C,D) for the five models of the relationship between DRCs and cataract illustrate the linear relationship between the effects of two DRCs with a high number of SNPs and the effects on cataract. It could be observed that the fit lines of the scatter plots for the five models are generally in the same direction, with most models having consistent slopes, and the intercept of the IVW model is close to zero.

### Sensitivity analysis

The Cochran Q test and I^2^ statistic evaluated heterogeneity of significant results (Supplementary Table S5), showing moderate heterogeneity in MR findings for oily fish intake and diet variation related to cataract (both P < 0.05). MR-Egger regression tested horizontal pleiotropy at the IV level; each indicator’s intercept P-values exceeded 0.05 and were near zero, indicating study causal inferences were unaffected by horizontal pleiotropy (Supplementary Table S6). A leave-one-out sensitivity analysis revealed no substantial changes in DRC effect estimates, suggesting result stability (Supplementary Table S7). The Steiger directionality test assessed the causal link from DRCs to cataract (Supplementary Table S8). Results indicated SNPs explained more variance for exposure than for the outcome, with a TRUE direction and P < 0.05, signifying significant and accurate directionality.

### Reverse MR analysis

**FIGURE 4:**
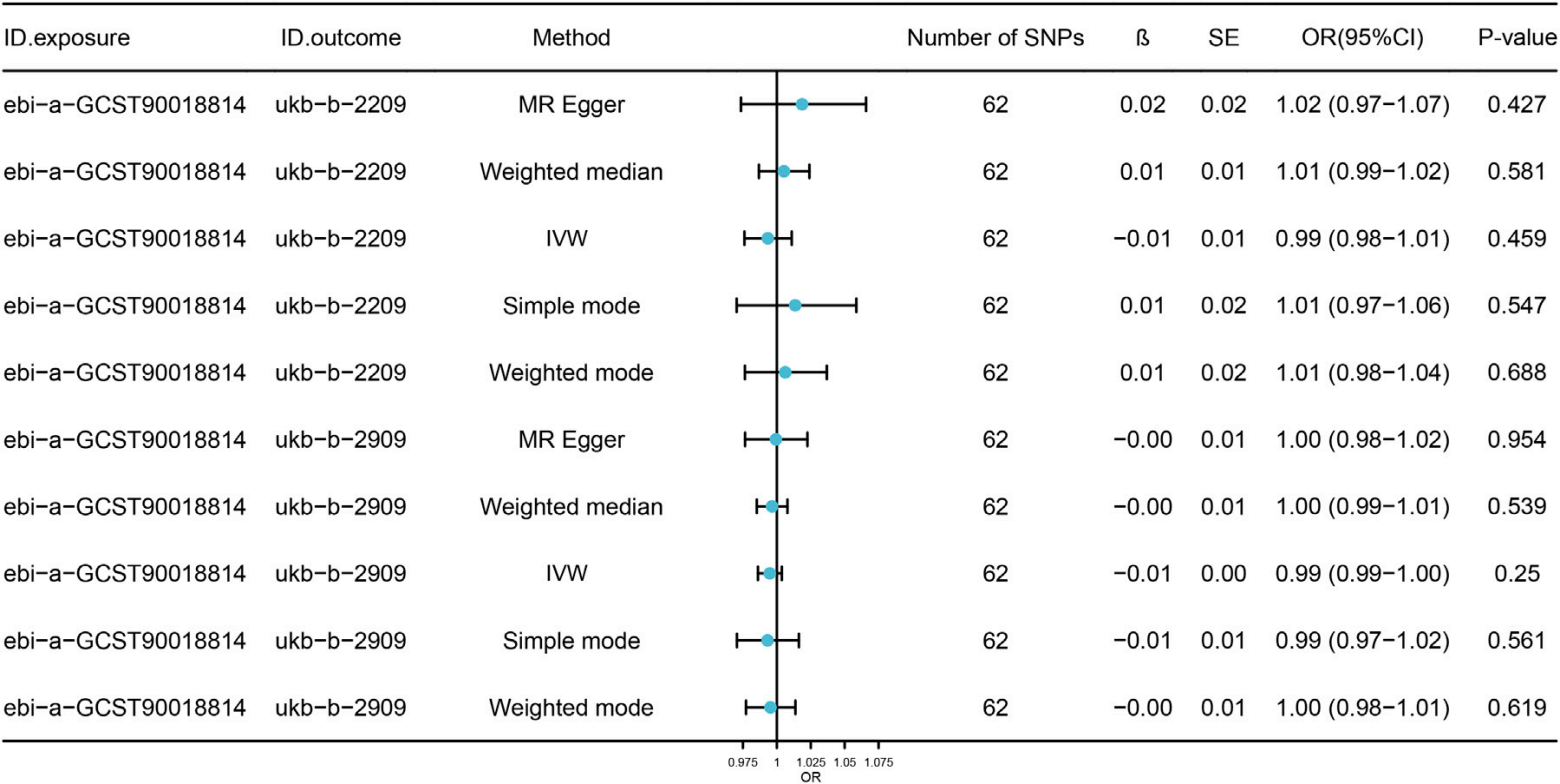
Model analysis results of reverse causality MR analysis of DRCs. The forest plots show the results of reverse causality analysis of multiple MR models for cataract and DRCs. The estimated effect values are presented as OR and 95%CI. The number of IVs used in each model, as well as the calculated Beta values and standard errors, are also shown. SNPs, single nucleotide polymorphisms; OR, odds ratio; SE, standard error; CI, confidence interval; β, Mendelian randomization analysis effect coefficient; IVW, Inverse variance weighted.

To assess reverse causality, we utilized cataract as the exposure and DRCs as the outcome, employing a GWAS of cataract with a SNP selection criterion of P < 5 × 10^−6^; SNPs in linkage disequilibrium were selected. The results of the reverse MR analysis indicate that cataract does not have a significant causal effect on DRCs (P > 0.05), as shown in Figure 4; Supplementary Table S9.

### MVMR analysis

We incorporated relevant risk factors for cataract, including diabetes, age, and smoking, into multivariate MR analysis along with two DRCs to assess the direct effects of these DRCs on cataract. We constructed models by pairing each of the cataract-related risk factors with the two DRCs to predict the relationship with the outcome, resulting in MVMR models. We obtained three meaningful MVMR models (Supplementary Table S10), and the results showed that these risk factors have significant causal effects on cataract in Models 1, 2, and 3 (P < 0.05).

### Mediation effect analysis

We first used the Steiger directionality test to determine the causal direction from two DRCs through metabolites (Supplementary Table S11). Results showed that for both DRCs, SNPs explained more variance for exposure than for outcome risk factors, with a TRUE direction and P < 0.05. We then assessed the mediation effect in MVMR models where the mediator significantly affected the outcome (Supplementary Table S12). In Models 1–4, DRCs significantly impacted cataract (P < 0.05), indicating a partial mediation effect via metabolites. Using univariate MR results, we determined the causal effect sizes of DRCs on metabolites. From the MVMR analysis, we obtained direct and indirect effect sizes of metabolites on cataract development. We calculated the mediation effect sizes of DRCs on cataract incidence through metabolites (Supplementary Table S13). The results show that oily fish intake has an indirect effect on cataract through total lipids in medium high-density lipoprotein with an effect size of −0.00419. The indirect effect accounts for 2.84% of the direct effect (|−0.00419/−0.05798|*100%).

## Discussion

Cataract is the leading cause of blindness globally, significantly impacting individuals' quality of life and socio-economic frameworks. However, the influence of dietary factors on cataract incidence and progression remains unclear. This study employed MR to investigate the causal relationships between dietary risk factors and cataract development. Our findings revealed a significant causal link between oily fish consumption and cataract formation, suggesting a protective effect. Oily fish is a rich source of long-chain omega-3 polyunsaturated fatty acids, such as eicosapentaenoic acid and docosahexaenoic acid [16]. Previous studies have shown that higher consumption of long-chain omega-3 fats, particularly in women, reduces the risk of cataract extraction surgery [17]. In contrast, dietary variation increased cataract risk. Monolithic dietary structures, like the Mediterranean diet and the Korean Balanced Diet, rich in unsaturated fatty acids, antioxidants, and plant-based products, have been shown to reduce ARC incidence [18–20]. Additionally, specific vitamin dosages may slow cataract formation [21]. The MVMR analysis indicated that diabetes, age, and smoking significantly impact cataract development. Diabetes approximately doubles cataract occurrence, while smoking cessation reduces its incidence, especially among the elderly [22]. Mediation analysis suggested that oily fish intake indirectly affects cataract risk through total lipids in medium HDL. Higher HDL cholesterol levels, influenced by long-chain n-3 PUFAs, are beneficial in reducing ARC risk [23].

In this two-sample MR study, the IVW method was primarily used to assess the causal relationship between dietary risk factors and cataract. The IVW model’s intercept was close to zero, and fitting curves were consistent across models, indicating a robust causal relationship [24]. The Weighted median and MR-Egger methods were used as supplementary tests. The MR-Egger regression intercept was close to zero, indicating no horizontal pleiotropy and good independence of selected SNPs.

To further validate the authenticity and robustness of our results, we conducted multiple sensitivity analyses, including Cochran’s Q test to assess heterogeneity, the MR-Egger method to detect horizontal pleiotropy, and leave-one-out analysis to confirm result consistency. Additionally, we performed Steiger directionality tests, which demonstrated that the variance explained by our selected IVs aligned with the expected causal direction, further strengthening the credibility of our causal inference. Simultaneously, in the reverse MR analysis, we evaluated the potential effect of cataracts on dietary-related characteristics. No significant causal relationship was observed, supporting the reliability of our findings. Furthermore, in MVMR analyses, we accounted for confounding factors such as diabetes, age, and smoking, which further confirmed the direct effects of dietary-related characteristics on cataract risk.

Heterogeneity testing revealed moderate heterogeneity in the MR analysis of oily fish intake and dietary variation, possibly due to gene-environment interactions. Gene-environment interactions may be an important contributing factor. For example, genetic variations in the metabolism of omega-3 fatty acids from fish differ across populations [25, 26], and dietary diversity can also lead to interindividual differences in nutrient absorption and utilization efficiency [27]. Additionally, environmental factors such as geographic location, cultural dietary habits, and lifestyle behaviors (e.g., smoking, alcohol consumption) may further exacerbate heterogeneity. For instance, specific genetic variants interact with fish intake in influencing metabolic disease risk-such as the association between fish consumption and TM6SF2 gene variants in modulating non-alcoholic fatty liver disease risk. Conversely, in other populations, environmental exposures may attenuate these effects [28]. Future studies should incorporate additional environmental covariates and gene-environment interaction terms to more comprehensively elucidate the sources of heterogeneity.

Our MVMR analysis identified smoking as an independent risk factor for cataracts, an association potentially mediated through chronic inflammation and epigenetic mechanisms. Emerging evidence indicates that smoking significantly upregulates GPR15 (an orphan receptor involved in immune regulation) and induces its hypomethylation [29]. Genome-wide transcriptomic analyses further demonstrate elevated GPR15 expression in peripheral blood of both male and female smokers, where this receptor promotes systemic chronic inflammation by modulating T-cell migration [30]. Given that lens oxidative stress and protein denaturation are central to cataract pathogenesis, GPR15-mediated sustained inflammatory microenvironments may accelerate lens epithelial cell damage. This molecular mechanism strongly aligns with our observed smoking-cataract association and concurrently provides a theoretical basis for the protective effects of dietary factors (e.g., ω-3 fatty acids in oily fish) through anti-inflammatory pathways. Future studies should explore GPR15’s potential as both a biomarker and therapeutic target for smoking-associated cataracts.

Notably, although obesity or body weight (e.g., BMI) is considered a potential confounder in the relationship between certain dietary factors and cataract risk, our analysis-based on publicly available GWAS databases-primarily centered on dietary-related exposures and outcomes, precluding direct adjustment for BMI or weight. However, the strength of MR lies in its ability to mitigate phenotypic confounding bias, including obesity, at the genetic level. The independence of our selected instrumental variables helps reduce potential bias arising from unadjusted weight-related factors. Furthermore, obesity is closely correlated with other included risk factors like diabetes, the effects of which are partially accounted for in the multivariable model. Future studies with larger samples and more comprehensive multivariable analyses may further elucidate the joint effects of weight/BMI and dietary factors on cataract risk, providing deeper insights into the complex interplay between diet, obesity, and cataract development. It is noteworthy that other modifiable lifestyle factors, such as BMI and physical activity, have also been implicated in cataract risk. However, the analytical framework of this study primarily focused on elucidating the causal relationship between dietary-related characteristics and cataracts, and thus did not conduct in-depth analyses of BMI, physical activity, or other related factors. Future research should further investigate the causal associations and underlying mechanisms linking BMI, physical activity, and other lifestyle factors to cataract risk, thereby strengthening the scientific foundation for comprehensive cataract prevention and control strategies.

The study’s limitations include a limited sample size, focus on European descent, and reliance on MR methods without wet-lab validation. Future research should include larger, multi-ethnic cohorts, longitudinal designs, and wet-lab experiments to verify the molecular pathways through which dietary factors influence cataract development.

In conclusion, this study provides new insights into the relationship between dietary risk factors and cataract, highlighting the protective role of oily fish and the potential risk associated with dietary variation. These findings offer a foundation for future prevention strategies and emphasize the need to explore the mediating roles of metabolites and their biological mechanisms.

## Data Availability

The original contributions presented in the study are included in the article/Supplementary Material, further inquiries can be directed to the corresponding authors.

## Footnotes

1
https://gwas.mrcieu.ac.uk/

## Abbreviations

MR, Mendelian randomization; DRCs, dietary-related characteristics; SNPs, single nucleotide polymorphisms; IVs, instrumental variables; MVMR, Multivariable Mendelian Randomization.
